# Bilateral Variation in the Origin and Course of the Vertebral Artery

**DOI:** 10.1155/2012/580765

**Published:** 2012-06-06

**Authors:** Aprajita Sikka, Anjali Jain

**Affiliations:** Department of Anatomy, Christian Medical College, Ludhiana, Punjab 141008, India

## Abstract

Understanding the great vessels of the aortic arch and their variations is important for both the endovascular interventionist and the diagnostic radiologist. An understanding of the variability of the vertebral artery remains most important in angiography and surgical procedures where an incomplete knowledge of anatomy can lead to serious implications. In the present case, a bilateral variation in the origin and course of vertebral artery was observed. The left vertebral artery took origin from the arch of aorta and entered the foramen transversarium of the fourth cervical vertebra. The right vertebral artery took origin from the right subclavian artery close to its origin and entered the foramen transversarium of the third cervical vertebra. The literature on the variations of the artery is studied and its clinical significance and ontogeny is discussed.

## 1. Introduction

Anatomical variation is defined as the normal flexibility in the topography and morphology of body structures [1]. Many or most variations are totally benign; some are errors of embryologic developmental timing or persistence of normally obliterated structures [2].

Understanding the great vessels of the aortic arch and their variations is important for both the endovascular interventionist and the diagnostic radiologist. An understanding of the variability of the vertebral artery remains most important in angiography and surgical procedures where an incomplete knowledge of anatomy can lead to serious implications. This has become more important in the era of carotid artery stents, vertebral artery stents, and therapeutic options for intercranial interventions [3].

 In angiographic and anatomic postmortem examinations, abnormal vertebral artery origins are incidental findings because, in most cases, they are clinically asymptomatic; nonetheless, these abnormalities are of diagnostic importance either prior to vascular surgery in the neck region or in cases of intravascular disease such as arteriovenous malformations or cerebral aneurysms, thrombosis, occlusion, arterial dissection, and potentially atherosclerosis [1, 4, 5].

The vertebral artery arises from the superior aspect of the subclavian artery, passes through the foramina of all cervical transverse processes except the seventh, curves medially behind the lateral mass of atlas, and then enters the cranium via the foramen magnum. At the lower pontine border, it joins its fellow to form the basilar artery. Occasionally, it may enter the bone at fifth, fourth, or seventh cervical transverse foramen [6].

An abnormal origin of the vertebral artery may favour cerebral disorders because of alterations in cerebral hemodynamics and predispose the patient to intracranial aneurysms [7].

## 2. Materials and Methods

During routine dissection of head and neck region in the dissection hall, a bilateral variation in the origin and course of vertebral artery in an adult female cadaver whose age was around 35 years was observed. The diameters of the two arteries were measured at the origin and where they entered the foramen transversaria with digital vernier calipers (in millimeters [mm]). The length of the arteries was measured with a thread and vernier calipers (in mm).

## 3. Results

**Figure 1:**
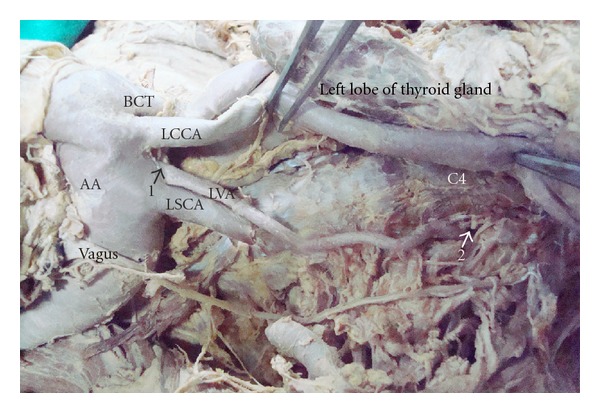
1: Origin of left vertebral artery, 2: Level where left vertebral artery enters foramen transversarium, LVA: Left vertebral artery, AA: Arch of aorta, BCT: Brachiocephalic trunk, LCCA: Left common carotid artery, LSCA: Left subclavian artery, C4: Fourth cervical vertebra.

The left vertebral artery took origin directly from the arch of aorta between the left common carotid artery and the left subclavian artery. The origin was posterolateral to the left common carotid artery, just proximal and anterior to the left subclavian artery (Figure 1). The artery was tortuous, crossed the left common carotoid artery posteriorly to come, and lie on its medial side. It then ascended paravertebrally and entered the foramen transversarium of the fourth cervical vertebra (Figure 1). Thereafter, it followed the normal course to enter the cranial cavity through foramen magnum.

**Figure 2:**
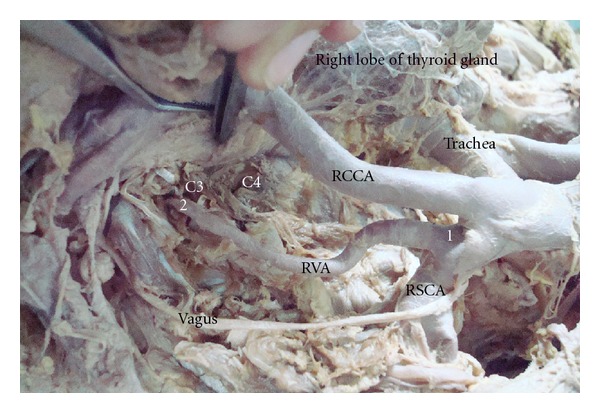
1: origin of right vertebral artery, 2: Level where right vertebral artery enters foramen transversarium, RVA: Right vertebral artery, RSCA: Right subclavian artery, BCT: Brachiocephalic trunk, RCCA: Right common carotid artery, C3: Third cervical vertebra, C4: Fourth cervical vertebra.

The origin of the right vertebral artery was also variable in the same cadaver. It took origin from the right subclavian artery at the junction of its origin from the Brachiocephalic trunk. It was dilated at its origin (Figure 2). It passed posterior to the right common carotid artery to come and lie medially. It then ascended to enter the foramen transversarium of the third cervical vertebra (Figure 2).

The length of the left vertebral artery from its origin to where it entered the foramen transversarium of C-4 was 91.69 mm where as that of the right from its origin to the foramen transversarium of C-3 was 78.35 mm. Though the left vertebral artery entered the 4th foramen transversarium and the right entered the 3rd, the greater length of the left could be attributed to its tortuosity.

The diameters of the left vertebral artery at origin and where it entered the foramen were 4.96 mm and 3.93 mm, respectively, whereas those for the right were 5.62 mm and 3.16 mm, respectively.

## 4. Discussion

A thorough understanding of anomalous vertebral arteries is paramount when performing both diagnostic and interventional angiography. Contrast enhanced MRA is becoming increasingly common, and with improved resolution, identifying pathology including ostial lesions of the great vessels and vertebral arteries will become more frequent [1].

Advances in imaging techniques and surgery, especially reconstructive and minimally invasive procedures, have necessitated a more accurate knowledge of the variability of the human body [5].

Maldevelopment anomalies of the vertebral arteries are generally considered very rare and, to date, have been described in single-case reports and in small series of patients with a single type of pathology [5]. Anomalous blood vessels are of common occurrence. They may be due (i) to the choice of unusual paths in the primitive vascular plexus, (ii) to the persistence of vessels normally obliterated, (iii) to the disappearance of vessels normally retained, and (iv) to incomplete development and to fusions and absorption of parts usually distinct [8].

The factors controlling the selection and differentiation of the appropriate channels in the plexuses and the elaboration of the structural characteristics of their walls are not completely understood. As stated earlier, it is known that genetic factors and local hemodynamic influences such as rate and direction of flow and pressure of the blood are both concerned in the establishment of final pattern [9].

The vertebral artery is an important vessel, which arises as a secondary development, on each side of the midplane, from a series of dorsal rami of dorsal intersegmental arteries belonging to the neck. These rami undergo longitudinal linkage just dorsal to the ribs (post costal anastomosis). All of the original stalks then atrophy except the most caudal one in the series. The resulting longitudinal vessel is the vertebral artery; it takes origin, along with the subclavian from the seventh intersegmental artery. The seventh cervical intersegmental continues as the left subclavian and hence as the distal part of the right [8].

A left vertebral artery of aortic origin may be because of the persistence of the dorsal division of the left 6th intersegmental as the first part of the vertebral artery instead of that of the left 7th intersegmental artery [5], which seems to be the cause of variation in our case.

The origin of the left vertebral artery from the arch of aorta has been documented by different authors with a range of 3.1%–8.3% [10].

The right vertebral artery may arise (a) from the first part of the subclavian, nearer than normal to the brachiocephalic (1% of cases) or to the anterior scalene muscle, (b) directly from the arch of aorta (3% of cases), (c) from the right common carotid, when the right subclavian arises from the aorta beyond the left subclavian, or (d) from the brachiocephalic trunk [10].

In the present case, the right vertebral arose from the first part of the right subclavian at the junction of its origin from the brachiocephalic trunk. Either vertebral artery may enter the foramen in the second through seventh cervical vertebra. When entering one of the higher vertebral foramina, the artery may lie behind the common carotid.

The vertebrals enter the sixth cervical foramen in 88% cases, seventh in 5%, and fifth in 7% cases [2]. In another study, the artery is reported to enter 6th, 7th, 5th, and 4th cervical vertebrae in 94.9%, 0.3%, 3.3%, and 1.6% cases, respectively [9]. According to Gray's anatomy, the artery enters the foramen transversarium of the 6th cervical vertebra in 90% cases, while those of 7th, 5th, 4th, and 3rd in 2%, 5%, 2%, and 1% cases, respectively [11].

 In a study by Bruneau et al., out of 500 vertebral arteries studied, they observed an abnormal level of entrance into foramen transversarium in 7% specimens (35 cases), with a level of entrance into the C3, C4, C5, or C7 foramen transversarium respectively in 0.2%, 1.0%, 5.0% and 0.8% of all specimens. Seventeen abnormalities were right sided and 18 were left sided. Thirty-one out of 250 patients had a unilateral anomaly and two had a bilateral anomaly [12]. The present case shows that a bilateral anomaly though the level of entrance is different on the right (C3) and the left side (C4).

In approximately 60% cases, the arteries are unequal in size. The left vertebral artery is often larger in size than the right, which is true in our case, except at the origin [5, 7].

 Without a thorough understanding of anomalous origins of the great vessels, angiography can be difficult or impossible. If the vertebral arteries are not identified in their normal position, this finding can be misinterpreted as the vessels being congenitally absent. This information is important for vascular or cardiothoracic surgical planning [1].

 Anomalous origins may lead to altered hemodynamics and predispose the patient to intracranial aneurysm formation. Therefore, in patients with these anomalies, a thorough search for coexisting aneurysms should be undertaken. Endovascular therapy can be performed before they present clinically as subarachnoid hemorrhages or by mass effect and, thereby, decrease morbidity and mortality [1].

Surgical procedures that would necessitate exposure of vertebral artery include repair of aneurysms, excisions of craniocervical junction masses, vertebral endarterectomy, vertebral artery bypass, and bony decompression of the vertebral artery. Also anatomical variations in vertebral artery if missed can lead to catastrophic sequelae in surgeries like atlantoaxial transarticular screw fixation, anterior cordectomy.

Ultimately, with respect to individual variations of the vertebral artery, a thorough knowledge of vertebrobasilar variations may improve the outcome of skull base and other head and neck operations and aid in the interpretation of imaging [5].

Advances in technology have increased our knowledge regarding different variations in our body and an awareness regarding them can help avoid unwanted complications during various interventions.

## References

[1] Satti SR, Cerniglia CA, Koenigsberg RA (2007). Cervical vertebral artery variations: an anatomic study. *American Journal of Neuroradiology*.

[2] Bergman RA, Afifi AF, Miyauchi R, Bergman RA, Afifi AF, Miyauchi R Opus II: cardiovascular system vertebral artery variations. *Illustrated Encyclopedia of Human Anatomic Variation*.

[3] Poonam, Singla RK, Sharma T (2010). Incident of anomalous origins of vertebral artery—anatomical study and clinical significance. *Journal of Clinical and Diagnostic Research*.

[4] Lemke AJ, Benndorf G, Liebig T, Felix R (1999). Anomalous origin of the right vertebral artery: review of the literature and case report of right vertebral artery origin distal to the left subclavian artery. *American Journal of Neuroradiology*.

[5] Shoja MM, Tubbs RS, Khaki AA, Shokouhi G, Farahani RM, Moein A (2006). A rare variation of the vertebral artery. *Folia Morphologica*.

[6] Standring S (2008). Figure 28.10. the level of the vertebral arteryinto the foramina transversaria of the cervical vertebrae. Vascular supply and lymphatic drainage. Head and Neck. *Gray’s Anatomy, the Anatomical Basis of Clinical Practice*.

[7] Patasi B, Yeung A, Goodwin S, Jalali A (2009). Anatomic variation of the origin of the left vertebral artery. *International Journal of Anatomical Variations*.

[8] Arey LB (1957). Development of arteries. The vascular system. *Developmental Anatomy. A Textbook and Laboratory Manual of Embryology*.

[9] Hamilton WJ, Boyd JD, Mossman HW (1972). Cardiovascular system. *Human Embryology, Prenatal Development of Form and Function*.

[10] Singla RK, Sharma T, Sachdeva K (2010). Variant origin of left vertebral artery. *International Journal of Anatomical Variations*.

[11] Williams PL, Bannister LH, Berry MM (1995). Cardiovascular system—subclavian system of arteries. *Gray’s Anatomy*.

[12] Bruneau M, Cornelius JF, Marneffe V, Triffaux M, George B (2006). Anatomical variations of the V2 segment of the vertebral artery. *Neurosurgery*.

